# *Notocotylus loeiensis* n. sp. (Trematoda: Notocotylidae) from *Rattus losea* (Rodentia: Muridae) in Thailand

**DOI:** 10.1051/parasite/2011181035

**Published:** 2011-02-15

**Authors:** K. Chaisiri, S. Morand, A. Ribas

**Affiliations:** 1 Department of Helminthology, Faculty of Tropical Medicine, Mahidol University Bangkok 10400 Thailand; 2 Department of Zoology, Faculty of Science, Kasetsart University Bangkok 10900 Thailand; 3 Institut des Sciences de l’Evolution, UMR 5554 CNRS-IRD-UM2, CC65, Université de Montpellier 2 34095 Montpellier France; 4 CIRAD, UR22 AGIRs, Campus International de Baillarguet 34398 Montpellier France; 5 Department of Population Biology, Institute of Vertebrate Biology, 16 Academy of Sciences of the Czech Republic 675 02 Konesin, Studenec 17 122 Czech Republic

**Keywords:** *Notocotylus*, Trematoda, Digenea, Notocotylidae, *Rattus losea*, lesser rice field rat, Thailand, *Notocotylus*, Trematoda, Digenea, Notocotylidae, *Rattus losea*, petit rat des rizières, Thaïlande

## Abstract

*Notocotylus loeiensis* n. sp. (Trematoda: Notocotylidae) is described from the cecum of the lesser rice field rat (*Rattus losea*), from Loei Province in Thailand with a prevalence of 9.1% (eight of 88 rats infected). The new species differs from previously described *Notocotylus* species mainly by the extreme prebifurcal position of the genital pore and the number of ventral papillae. This is the first description at the species level of *Notocotylus* from mammals in Southeast Asia.

## Abstract (fr)

*Notocotylus loeiensis* n. sp. (Trematoda : Notocotylidae) du caecum du petit rat des rizières (*Rattus losea*) a été observé chez huit rats sur 88 (9,1 %) dans la province de Loei en Thaïlande. Cette nouvelle espèce diffère de celles de *Notocotylus* décrites précédemment, principalement par la position prébifurcale extrême du pore génital et par le nombre de papilles ventrales. Il s’agit de la première description du niveau d’espèce *Notocotylus* chez un mammifère en Asie du Sud-Est.

## Introduction

The trematode genus *Notocotylus* is cosmopolitan, with more than forty species parasitizing aquatic birds, and a few species also described from mammals. Of the latter group, *N. neyrai* Castro, 1945 was reported from *Arvicola sapidus* Miller, 1908 in Europe (Feliu *et al.*, 1986), *N. fosteri* Kinsella & Tkach, 2005 from the rice rat *Oryzomys palustris* (Harlan, 1937) in Florida, and *N. urbanensis* (Cort, 1914) from *Ondatra zibethicus* (Linnaeus, 1766) in Nebraska. In Australia, *N. johnstoni* Cribb, 1991 and *N. imbricatus* (Looss, 1893) were reported from the Australian water rat *Hydromys chyrsogaster* Geoffroy, 1804 (Cribb, 1991). In China, *Notocotylus ratti* (Yie *et al.*, 1956) was described from *Rattus rattus alexandrinus* (Geoffroy, 1830). All species of this genus are associated with wetland habitats as emphasized by Kinsella & Tkach (2005), where the life cycle involves aquatic gastropods as intermediate hosts (Thi Le, 1986).

Thi Le (1986) reported larval stages of *Notocotylus intestinalis* (Tubangui, 1932) from two species of fresh water gastropods (*Alocinma longicornis* and *Parafossarulus striatulus*) in Vietnam but with no record of the final host. Nguyen Thi Le (1991) found *N. intestinalis* (Tubangui, 1932) in the domestic chicken, *Gallus domesticus*. Adam *et al.* (1993) studied *Notocotylus* sp. lophocercous cercariae from the mollusc *Bithynia siamensis goniomphalus* (Prosobranchia: Bithyniidae) in Thailand. Finally, Eduardo & Gaddi (2003) reported new records of *Notocotylus* spp. from wild birds in the Philippines: *N. intestinalis* from the greater painted-snipe *Rostratula benghalensis benghalensis*, *Notocotylus pacifer* (Noble, 1933) from the common moorhen *Gallinula chloropus lozanoi* and *N. naviformis* (Tubangui, 1932) from the whistling-duck *Dendrocygna arcuata arcuata*. Knowledge on adult *Notocotylus* from mammals in Southeast Asia is limited to Pham *et al.* (2001), who reported an unspecified member of the genus from two rodent species: the rice field rat *Rattus argentiventer* (Robinson & Kloss, 1916) and the lesser rice field rat *Rattus losea* (Swinhoe, 1871) captured in Bac Ninh Province (Vietnam). To our knowledge, there are no reports of *Notocotylus* identified to species from mammals in Southeast Asia.

The lesser rice field rat (*R. losea*) is widely distributed from Taiwan, Hainan, and Fukien in China to Vietnam, Laos PDR and Thailand (Marshall, 1988). This rodent is mainly found in rice fields and other cultivated land, where it burrows into the ground, but it has also been found in grassy areas in natural pine forests at 850 m in Thailand and in coastal mangrove forest (Marshall, 1988 cited by Corbet & Hill, 1992). In the course of the CERoPath project, rodents were captured in Loei Province, in the northeast of Thailand on February 2008. A species of *Notocotylus* that did not match any described species was recovered from *R. losea*.

## Materials and Methods

Rodents were trapped in Loei province (Latitude: 17°, 490219; Longitude: 101°, 688536), Thailand, in 2008. Rats were collected near fresh water habitats in lowland paddy fields and soybean fields. These habitats were closely located by the branch of irrigation system and approximately one kilometer far from main river. Rodents were dissected after capture and their guts isolated and preserved in alcohol for future dissection at the laboratory.

Intestinal tracts of 88 *R. losea* were examined for helminths. Trematodes were isolated and preserved in alcohol, and later were post-fixed in Bouin’s solution, stained in Semichon’s acetocarmine and mounted in Canada balsam. Some unstained specimens were also mounted in Canada balsam, and several specimens were dissected to study the eggs. Measurements are in micrometres and are listed as the holotype, followed by the range of the paratypes and the mean of five paratypes in parentheses. Drawings were made with the aid of a camera lucida.

## Description: *Notocotylus Loeiensis* n. sp. (Figs 1-4)

**Figs 1-4.**
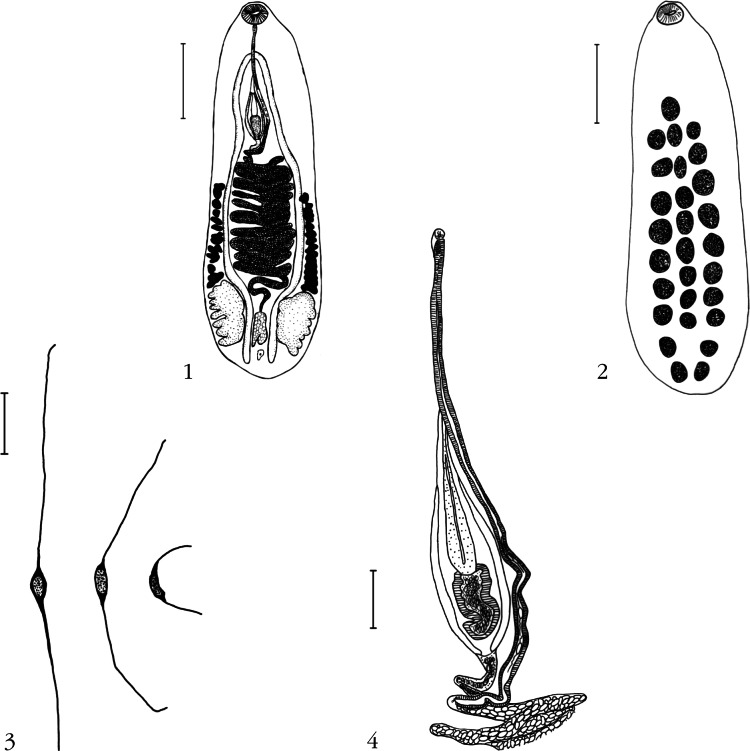
*Notocotylus loeiensis* n. sp. 1. in ventral view (scale bar: 0.5 mm); 2. Distribution of ventral papillae (scale bar: 0.5 mm); 3. Eggs (scale bar: 10 μm); 4. Detail on genitalia (scale bar: 0.1 mm).

Type host: lesser rice field rat, *Rattus losea* (Swinhoe, 1871) (Rodentia: Muridae).

Site: cecum.

Type locality: Muang District, Loei Province, Thailand. (Latitude: 17°, 490219; Longitude: 101°, 688536).

Type specimens: type specimens were deposited in the Museu de Ciències Naturals de Barcelona (Barcelona Natural Science Museum, Catalonia, Spain), codes of holotype MZB 2009-3134,and five paratypes MZB 2009-3135 to 2009-3139. Additional individuals are deposited in the “Southeast Asia small mammals helminth collection” in the Laboratory of Parasitology of the University of Barcelona (Spain).

Collection dates: February 2008.

Prevalence and intensity of infection: prevalence of 9.1% (8 of 88). Intensity 2-20 (mean 82.5).

Etymology: this species is named for the Loei Province, where this trematode was collected.

### Description

Notocotylinae Kossack, 1911. Based on holotype and five paratypes mounted *in toto* with characters of genus. Body 2,112 (1,678-2,461) long and 770 (834-659) maximum width at about level of ovary. Tegument unspined. Total number of ventral papillae 27-31; lateral and medial rows all contain 9- 11 papillae (usually 9-10). Second papilla of middle row at same level as first papilla of lateral rows (see Fig. 2). Cercarial eye-spot pigment scattered in forebody. Oral sucker 124.28 (130.56-110.8) long, 160.43 (166.4-151.04) wide. Oesophagus 234 (267-154) long, bifurcating into two caeca, which curve between ovary and testes, and terminate blindly 103.0 (43.52- 151.04) or 4.90% (1.78-7.27%) of body length from posterior end of body, anterior or posterior to end of testes. Testes opposite, deeply lobed, longer than broad 415 (298-494) long, 219 (164-267) wide. Cirrus sac median, straight, 722 (607-803) long, broad posteriorly and sharply narrowed anteriorly, containing saccular internal seminal vesicle and pars prostatica. Cirrus not everted in available specimens. Common genital pore ventro-medial, considerably in front of intestinal bifurcation reaching posterior margin or overlaying oral sucker ventral to beginning of oesophagus. Ovary median, between testes, never entire, bilobed posteriorly, 188 (144-236) long, 107 (82-144) wide. Vitelline reservoir immediately anterior to ovary. Vitellarium formed of two lateral groups of 11-16 bilobed to multilobed follicles extending from 23.12- 27.13 (24.76) % of body length from posterior end to 44.85-66.22 (50.60) % of body length from anterior end. Uterus extending anteriorly from ovary in 12-15 major, laterally directed coils, with 2-4 coils anterior to vitelline follicles; some uterine coils slightly exceeding caeca but are contained by vitelline follicles. Metraterm muscular, sinistral at level of cirrus pouch (see Fig. 4). Eggs operculate bearing single long, filament at each pole; egg capsule length 16-19 (18) and 10-11 (10) wide (n = 20), each filament of variable size 29-76 (44) long. Excretory pore opening dorsally at about level of posterior end of testes; excretory vesicle saccular.

## Discussion

Kinsella & Tkach (2005) considered 49 species of the genus *Notocotylus* to be valid. To this number should be added *N. biomphalariae* (Flores & Brughi, 2005). The species *N. gippyensis* and *N. tadornae* should be removed from this list, as these two have only one row of ventral papillae and thus must be transferred to the genus *Uniserialis* according to Barton & Blair (2005). The number is then reduced to 48 valid species.

Of these 48 species, only *N. aegyptiacus* (Odhner, 1905), *N. fosteri*, *N. johnstoni*, *N. mamii* (Hsu, 1954), *N. naviformis* Tubangui, 1932, *N. skrjabini* Ablasov, 1953 [synonym: *N. breviserialis* (Stunkard, 1967)], and *N. vinodae* have a prebifurcal genital pore. Of these species, only *N. fosteri* has a genital pore as far anterior as *N. loeiensis* (ventral to the posterior margin of the oral sucker). However, *N. fosteri* has a higher number of ventral papillae (10-13) *versus* (9-11) in *N. loiensis* n. sp. The number of previtelline uterine loops is greater (5-7) in *N. fosteri* than in *N. loeiensis* n. sp. (2-4), the ovary in *N. loeiensis* n. sp. is strongly lobulated (Fig. 1) while the ovary in *N. fosteri* is usually entire, or occasionally bilobed posteriorly.

With respect to ventral glands, *N. breviserialis* and *N. skrjabini* have only 4-5, *N. vinodae* has 15-16 and *N. aegyptiacus* has 12-14 (Kinsella & Tkach, 2005). In *N. johnstoni*, the number of papillae in the median row (9-11) usually is one or two less than the number in the lateral rows (10-13).

Of species reported from birds in Southeast Asia, the new species differs from *N. intestinalis* and *N. pacifier* which both have a postbifurcal genital pore, and from *N. naviformis* by the number of ventral glands (13 in medial row in *N. naviformis*) and by the distance between the medial gland and the lateral (2.5 gland length in *N. naviformis* and less than one gland length in *N. loeiensis* n. sp.) (see Fig. 2).

Of species reported from southern China, *N. loeiensis* sp. n. differs from *N. mammi* from Canton, which was obtained from experimental infections of laboratory rabbits by metacercariae. The glands of the middle row are not at the same level as the laterals as observed in *N. loeiensis* n. sp. The ovary of *N. mammi* has 2-8 lobes (usually 4-6) *versus* being bilobulated in *N. loeiensis* n. sp. The only species found naturally in Chinese mammals is *N. ratti*, which differs from *N. loeiensis* n. sp. in having only 5 to 6 ventral glands in the medial row, and has a postbifurcal genital pore (Yie *et al.*, 1956). Three other species have been reported in birds from Jiangxi Province by Qiongzhang (1992): *N. polylecithus* differs in having twice as many ventral glands in each row (27-28 in the lateral rows and 24-25 in the middle row); *N. urbanensis* has a postbifurcal genital pore, 13-14 glands in the lateral rows, and 12-13 glands in the middle row; and *N. thienemanni* has two glands of the middle row anterior to the lateral rows instead of one as in *N. loeiensis* n. sp, and 10 glands in the lateral rows and 14 in the middle row.

The great variability in size of polar filaments from eggs (Fig. 3), also noted by González-Castro (1945), suggests that this character should not be used in species diagnoses.

In conclusion, *N. loeiensis* n. sp. is the first species of *Notocotylus* described from Southeast Asian mammals.
